# Living Donor Intestinal Transplant: Indication, Techniques, Surgical Complications, and Outcomes in Recipients and Donors: A Systematic Review

**DOI:** 10.1155/joot/4030621

**Published:** 2026-05-31

**Authors:** Adil Nusair, Saad Bin Yousuf, Ahmed Adham R. Elsayed, Marc D. Basson

**Affiliations:** ^1^ College of Medicine, Northeast Ohio Medical University, Rootstown, Ohio, 44272, USA, neomed.edu; ^2^ Department of Surgery, Northeast Ohio Medical University, Rootstown, Ohio, 44272, USA, neomed.edu; ^3^ Department of Biomedical Sciences, Northeast Ohio Medical University, Rootstown, Ohio, 44272, USA, neomed.edu

**Keywords:** intestinal failure, intraoperative complications, living donor intestinal transplant, postoperative complications, risk factors

## Abstract

**Background:**

Living donor intestinal transplantation (LDITx) is an alternative option to cadaveric transplants as a last resort in treating intestinal failure. There are limited data on LDITx outcomes. This systematic review evaluates LDITx in terms of indications, contraindications, surgical complications, and patient outcomes.

**Methods:**

A comprehensive search was conducted across PubMed, Cochrane Library, Virtual Health Library, and Web of Science databases following the PRISMA guidelines. After filtration, data were systematically extracted from all relevant observational studies, case series, and case reports. Quality assessments were performed using the CARE guideline for case reports and case series, while STROBE was used for observational studies.

**Results:**

Fifty‐four studies were included, comprising 11 observational studies, 9 case series, and 34 case reports. The main indications of LDITx were the availability of a compatible donor and the urgency of the recipient’s clinical condition. The standard surgical technique involves segmental ileal resection and transplantation. Postoperative complications included infections, ischemic events, and vascular complications. Recipients had either manageable complications or no complications. About 21.2% of the population experienced at least one episode of rejection, with an overall mortality rate of 23%. Many cases reported successful TPN weaning and good outcomes for the recipients and donors.

**Conclusion:**

In patients with chronic IF complicated by severe TPN‐related morbidity or underlying pathologies causing SBS, LDITx represents an effective treatment option and an alternative to the more conventional cadaveric intestinal transplantation, bypassing problematic time sensitivity and donor availability.

## 1. Introduction

First named in 1980, intestinal failure (IF) is characterized by reduced capacity for gastrointestinal absorption that requires macronutrient or fluid supplementation [1, 2]. Several treatment modalities exist, including long‐term parenteral nutrition [3] and lengthening surgery for patients reaching the plateau of intestinal adaptation [4], but intestinal transplantation (ITx) remains the last option in complicated cases [1]. There are two standardized forms of ITx, using either a cadaveric or living donor. Living donor intestinal transplantation (LDITx) is less commonly performed because of the risks to the donor and potentially inferior performance of the segmental graft [5]. This systematic review aims to evaluate LDITx in terms of indications, contraindications, surgical complications, and short‐ and long‐term outcomes of the procedure.

IF can be categorized as acute or chronic IF [1]. Acute IF is reversible within 6 months of onset, while chronic IF has long‐lasting consequences that usually require long‐term total parenteral nutrition (TPN) [6]. Long‐term TPN complications, including liver dysfunction, mucosal atrophy, and central vein thrombosis due to catheter‐related complications, may ultimately preclude continued therapy [7]. When such complications occur, the only remaining option is ITx [8, 9]. Since the first ITx attempted in 1967 and the first LDITx occurring shortly after in 1969, advancements in the field of immunology and transplant surgery have substantially increased safety and success rates [10–12].

Between 2005 and 2014, there was a recorded 55% increase in short bowel syndrome (SBS)–related hospitalizations, rising from 4037 to 6,265, which can be attributed to factors, such as improved survival rates of premature infants and patients, which makes for a larger population in need of extensive intestinal surgery, thus increasing the prevalence of IF [13]. In the United States, intestinal transplants are the least common form of organ transplantation, as they are often considered the most difficult, so outcome information has been limited [14]. Given the higher rate of complications and failure associated with ITx compared to other organ transplants, and since most available literature focuses on cadaveric donors, further investigation into LDITx is necessary to determine whether its benefits outweigh the risks [13]. This systematic review aims to analyze existing literature on LDITx to evaluate its potential advantages, complications, and outcomes. In so doing, it seeks to support clinical decision‐making, improve prospects for patients in need of an intestinal transplant, and provide valuable information for future research.

## 2. Materials and Methods

### 2.1. Search Strategy

This systematic review adheres to the Preferred Reporting Items for Systematic Reviews and Meta‐Analyses (PRISMA) guidelines. This search was performed on February 22, 2026, covering all the articles from inception to the date of the search within PubMed, Cochrane Library, Virtual Health Library, and Web of Science databases. The main keywords used were living donor intestine transplant, postoperative complications, indication, contraindication, and outcomes, or subsequent synonyms or MESH when available (See Supporting Table 1). Two independent reviewers filtered the obtained articles from the search by title and abstract, followed by a full‐text review based on the research question (What are the indications, surgical techniques, complications, and outcomes associated with LDITx?) and the eligibility criteria. Any disagreements between reviewers were discussed and rationalized between one another to come to a mutual consensus. Any sustained disagreements were referred to the senior author for a decision. No protocol was registered.

### 2.2. Eligibility Criteria

This review included articles that discussed LDITx, which covered indications, contraindications, surgical complications, or long‐term outcomes.

All articles that did not address outcomes for LDITx were removed during the initial filtration. Non‐English articles, nonhuman studies in animals or in vitro, and articles that were not original, including systematic reviews, literature reviews, and editorials, were all removed.

### 2.3. Data Extraction

Upon completion of the filtration process, all articles relevant to our question were imported into Zotero for the extraction of the characteristic and demographic data by the following subheadings: Author, year, study design, number of populations, separated into the total and relevant population in the given study, age, gender, and patient characteristics.

Data extraction for outcomes was performed using the following subheadings: author, year, condition which outlined any prior health concerns of the recipient, indication, recipients surgical technique, recipient postoperative complication and incidence, donor surgical technique, donor postoperative complication and incidence, patient rejection and incidence, number of rejection episodes per patient, number of rejection episodes which represented the number of rejection incidences per patient, recipient mortality and incidence, recipient length of stay, other outcomes which mainly assessed any outstanding long‐term postoperative finding proposed in each article that proved a merit mention for the recipients, and risk factors.

### 2.4. Aggregated Calculated Incidence

All aggregated percentages throughout this article were calculated relative to the total relevant population of 287 recipients. Adjustments were made to the overlapping population found within the final articles included in this review. Any articles that made no explicit mention of postoperative complications, rejection, or mortality also had their populations excluded from the section‐specific calculations. Articles that stated none of the patients included exhibited the parameter of interest were still retained in the analysis.

### 2.5. Reporting Quality Assessment

The EQUATOR network guidelines were utilized to assess all the articles. The Strengthening the Reporting of Observational Studies in Epidemiology (STROBE) guideline was used to review all observational studies, and the Consensus‐based Clinical Case Reporting (CARE) guideline was used to review all case reports and case series. Risk of bias (ROBINS‐I): All included observational (nonrandomized) studies were assessed using ROBINS‐I across seven domains, with overall judgment based on the worst‐domain rating; disagreements were resolved by consensus and the senior author if needed (Supporting Table 3).

## 3. Results

### 3.1. Search Results

**FIGURE 1:**
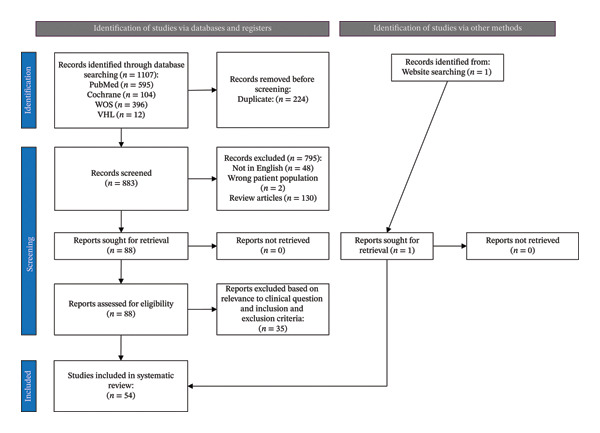
Identification of studies via databases and registers. PRISMA flow diagram depicting the identification, screening, eligibility, and inclusion process for studies used in the systematic review.

The initial search produced 1107 articles, of which 224 duplicates were removed to produce 883 articles. After two independent reviewers went through the screening process based on title, abstract, and full‐text review based on the eligibility criteria, 53 articles met the eligibility criteria. An additional article was identified through a website search, bringing the total articles to 54 (Figure 1) [5, 6, 15–66].

### 3.2. Characteristics and Demographics

The articles ranged in publication date from 1996 [64] to 2024 [5] and included 11 observational studies, 9 case series, and 34 case reports. The total number of patients included was 4,437, of which 287 were related to the research question. The largest study population was 61 [6], and the smallest was 1. The youngest patient was seven months old [33], whereas the oldest was 57 years old [6]. The most prominent patient characteristics were SBS, irreversible IF, and TPN dependency. Three articles utilized the same patient population with information that supplemented each other, but the population was only counted once [5, 16, 27]. The same exception was also made for another pair of articles [25, 60]. Another set of three included articles had overlapping patient populations, and all repeated patients were removed [58, 61, 67] (Table 1).

**TABLE 1:** Characteristics and demographics.

Author, year	Study design	Number of populations	Age	Gender	Patient characteristics
Wu et al., 2024 [5]	Observational study	Total: 54 LDITx: 54	—	—	All on TPN

Testa et al. 2008 [17]	Observational study	Total: 4 LDITx: 4	11–24 months (mean 15.5)	M: 2 F: 2	End‐stage liver and intestinal failure

Testa et al., 2005 [18]	Case report	1	1	M: 1	Prior LDIT that was rejected

Fan et al., 2015 [19]	Case report	1	16	F: 1	Severe bowel infarction

Uemoto et al., 2002 [20]	Observational study	Total: 3 LDITx: 3	2 y 6 m, 4 y 5 m, 3 y 5 m	M: 2 F: 1	Short bowel syndrome

Benedetti et al., 2001 [21]	Case report	1	27	M: 1	Short gut syndrome

Berney et al., 2004 [22]	Case report	1	13	M: 1	Short gut syndrome

Ueno et al., 2018 [23]	Observational study	Total: 27 LDITx: 13	0–35	—	Short gut syndrome [9], motility disorders [15], and retransplant cases [3]

Okada et al., 2002 [24]	Case report	1	16	M: 1	Microvillus inclusion disease had been receiving long‐term TPN for persistent diarrhea since birth, and the donor was the grandmother

Tesi et al., 1997 [25]	Observational study	Total: 2 LDITx: 2	26, 29	M, F	Both on long‐term TPN, female had Gardner’s syndrome, and male had intestinal dysmotility

Wu et al., 2022 [5]	Observational study	Total: 40 LDITx: 40	27.3 ± 16.4	13:12 ratio?	All on TPN

Garcia Aroz et al., 2017 [26]	Observational study	Total: 10 LDITx: 10	(9–156 years) 18 months med	5 M, 5 F	Irreversible intestinal failure, one or more complications, minimum weight of 8 kg

Wu et al., 2022 [27]	Observational study	Total: 40 LDITx: 40	—	—	Irreversible intestinal failure

Ceulemans et al., 2023 [6]	Comparative study	Total: 4156 LDITx: 63	(0.6–57 years) (20 med)	37 M, 26 F	SBS, motility disorder, tumor, mucosal defect, etc.

Ueno et al., 2023 [28]	Observational study	Total: 42 LDITx: 13	—	—	Intestinal failure due to different causes

Khan et al., 2021 [29]	Case report	1	55	F	Short bowel syndrome

Chaubal et al., 2021 [30]	Case report	1	9	M	Infected with COVID‐19

Noory et al., 2019 [31]	Case report	1	44	F	Morbidly obese

Benedetti et al., 2006 [32]	Observational study	Total: 11 LDITx: 11	2–50 years (26 avg)	7 M, 4 F	All had irreversible IF and ultra‐short bowel syndrome

Gangemi et al., 2009 [33]	Observational study	Total: 10 LDITx: 10	7–48 months (17 mean)	4 M, 6 F	Intestinal failure

Testa et al., 2004 [34]	Observational study	Total: 9 LDITx: 9	—	—	Irreversible intestinal failure due to gastroschisis or SBS

Ueno et al., 2013 [35]	Observational study	Total: 14 LDITx: 10	—	—	Short bowel syndrome [7], intestinal functional disorder [2], and retransplant [1]

Yue et al., 2023 [36]	Case report	1	29	F	Short bowel syndrome

Ji et al., 2009 [37]	Case report	Total: 4 LDITx: 4	18, 15, 23, 17 years	3 M, 1 F	Short bowel syndrome

Wu et al., 2017 [38]	Case report	1	18	M	Short bowel syndrome

Raofi et al., 2008 [39]	Case report	1	1	F	GI dysmotility

Grevious et al., 2009 [40]	Observational study	Total: 5 LDITx: 5	9–24 months (15.4 mean)	2 M, 1 F	Irreversible intestinal failure

Kumaran et al., 2012 [41]	Case report	1	43	M	Bowel ischemia

Schena et al., 2006 [42]	Case report	1	33	M	Asthmatic with gastrointestinal necrotizing vasculitis

Fujimura et al., 2021 [43]	Case report	1	11	M	Intestinal motility disorder

Li et al., 2008 [44]	Case reports	3	15–18 (15, 17, 28)	M	Short gut syndrome

Cicalese et al., 2001 [67]	Observational study	Total: 3 LDITx: 3	27–46 (27, 29, 46)	2 M, 1 F	Irreversible intestinal failure (short bowel syndrome)

Holterman et al., 2003 [45]	Case report	1	4	M	Profound malnutrition (SBS) and advanced TPN‐induced liver failure

Lee et al., 2004 [46]	Case report	1	57	F	Short bowel syndrome

Gruessner et al., 1997 [47]	Case report	Total: 1 LDITx: 1	16	M	Paraplegic w/severe TPN complications: no vascular access, recurrent infections, and intermittent liver dysfunction

Chang et al., 2016 [48]	Case series	Total: 3 LDITx: 3	2–56 (2, 48, 56)	2 F, 1 M	Hirschsprung disease and mesenteric vessel thrombosis

Qian et al., 2007 [49]	Case report	Total: 1 LDITx: 1	15	M	SBS from volvulus necrosis; subtotal enterectomy

Panaro et al., 2004 [50]	Case report	1	4	M	SBS from gastroschisis

Apichai et al., 2009 [51]	Case report	Total: 1 LDITx: 1	14 months	F	36‐week C‐section; gastroschisis w/intestinal volvulus; chronic liver failure (TPN cholestasis); multiple infections (peritonitis)

Ishii et al., 2006 [52]	Case reports	Total: 2 LDITx: 2	14, 27 (14–27)	M,F	TPN‐dependent SBS w/hypoganglionosis, massive enterectomy (volvulus), ABO identical, negative crossmatch, and CMV+

Song et al., 2005 [53]	Case report	Total: 1 LDITx: 1	18	M	Short gut syndrome

Benedetti et al., 2004 [54]	Case reports	3	27–30	M	Trauma‐induced short bowel syndrome

Wada et al., 2005 [55]	Case reports	Total: 2 LDITx: 2	14, 27 (14–27)	M,F	TPN‐dependent SBS w/hypoganglionosis, massive enterectomy (volvulus), ABO identical, negative crossmatch, and CMV+

Wang et al., 2005 [56]	Case report	Total: 1 LDITx: 1	17	M	Short bowel syndrome

Tzoracoleftherakis et al., 2002 [57]	Case report	1	28	M	Extensive abdominal injury from a close‐range shotgun blast

Cicalese et al., 2002 [58]	Case series	Total: 2 LDITx: 2	—	—	—

Morel et al., 2000 [59]	Case reports	Total: 1 LDITx: 1	13	M	Short gut syndrome, extensive resection (appendectomy), midgut volvulus, debilitating diarrhea, and failure to thrive

Jaffe et al., 1997 [60]	Case reports	Total: 2 LDITx: 2	26–29 (26,29)	F,M	10 cm proximal jejunum remaining (retroperitoneal desmoid tumor resection), TPN (21 months, 4 years), acquired ganglioneuropathy, dysmotility syndrome (pseudo‐obstruction), gastrostomy, and intact intestine except for resected terminal section

Cicalese et al., 2002 [61]	Case reports	Total: 1 LDITx: 1	27–30	M	Total enterectomies, IF; trauma (2 gunshot wounds, 1 motor vehicle accident)

Kim et al., 2012 [62]	Case report	Total: 1 LDITx: 1	3	F	Extended total aganglionosis, resection of entire colon, and most small bowel

Asham et al., 2006 [63]	Case report	Total: 1 LDITx: 1	44	F	Short gut syndrome and multiple bowel resections (familial polyposis)

Kuo et al., 1996 [64]	Case report	Total: 1 LDITx: 1	32	M	Recurrent retroperitoneal desmoid, involving proximal mesenteric artery/vein, tumor enlargement causing intestinal obstruction, refused chemotherapy, and prolonged TPN postresection

Wu et al., 2021 [65]	Observational study	Total: 36 LDITx: 19	35	F, M	Short gut syndrome

Beier et al., 2008 [66]	Observational study	Total: 11 LDITx: 11	25.55 (±) 18.04 months	54.55% Male	Gastroschisis, volvulus, rejection of first SB graft, cholestasis, graft loss (PTLD), megacystis microcolon, and necrotizing enterocolitis

### 3.3. Summary of Outcomes

The most common conditions were gastroschisis [26, 45], intestinal atresia [20], and incipient cholestasis [50, 57, 66]. The most prominent indication for LDITx was having a compatible donor available, typically an available parent, and time sensitivity [16, 27, 30]. Standard LDITx (segmental ileal graft procurement and transplantation with vascular anastomoses and restoration of intestinal continuity; see Sections 3.3.3 and 3.3.4) was the main surgical technique for recipients, whereas standard segmental ileal resection was the primary surgical technique used on the donor [6, 15, 16]. Recipient postoperative complications included ischemia (2.2%), infection (2.1%), and vascular complications (0.7%) [19, 41, 63], while some reported no postoperative complications (Supporting Table 2). The donor population experienced irregular bowel movements, causing diarrhea postoperatively [38, 58, 61, 67]. Rejection occurred at least once in 21.2% of the recipients, with the majority having only one rejection episode. The mortality rate was 23%. The patient with the shortest length of stay was 11 days [63], and the longest was 293 days [17]. Some unique risk factors included Hirschsprung disease [48], cholestatic jaundice [45], and physical/mental retardation [20, 52] (Supporting Table 2).

#### 3.3.1. Indication

**FIGURE 2:**
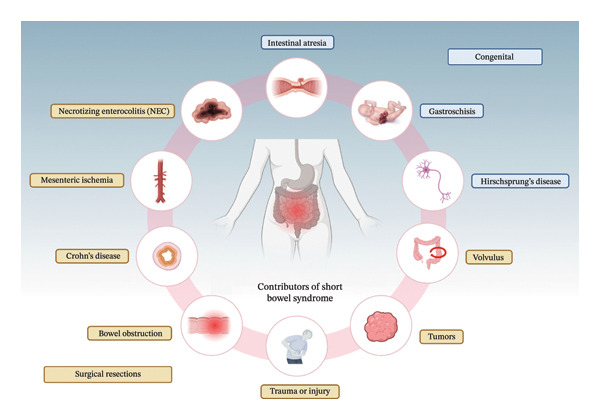
Contributors of short bowel syndrome (SBS). Divided contributors by congenital and surgical resection implications. Created in BioRender. Basson, M. (2025) https://BioRender.com/vps33fv.

All recipients undergoing LDITx had chronic IF, with most displaying severe SBS arising from disorders including Hirschsprung’s disease, bowel obstruction, and mesenteric ischemia (Figure 2). [68]. The main indications for performing LDITx were either time sensitivity due to life‐threatening TPN‐related complications or the availability of a compatible living donor with HLA matching [30, 38]. Some patients elected to undergo LDITx before the development of TPN‐related complications [16]. Some donors were identical twins, offering both availability and key benefits, including avoidance of immunosuppression and its associated side effects [9]. In some Asian countries (China, Japan, and Korea), cadaveric organ donation is less common than in Western countries due to cultural and religious beliefs, which has contributed to the increased use of LDITx [10]. In the United States, only 1.42% of intestinal grafts are obtained from living donors [11].

#### 3.3.2. Contraindication

The main contraindication to performing LDITx is concern for donor safety due to possible postoperative complications or long‐term effects of the segmental resection on the donor bowel, including potential sequelae later in life [9]. Potential risks, such as hemorrhage or long‐term small bowel obstruction, can also affect donor safety [27]. As recently as 2022, less than 3% of ITx utilized living donors, which may be partially attributed to the lack of a standardized surgical protocol for LDITx [10].

#### 3.3.3. Recipient Surgical Technique

**FIGURE 3:**
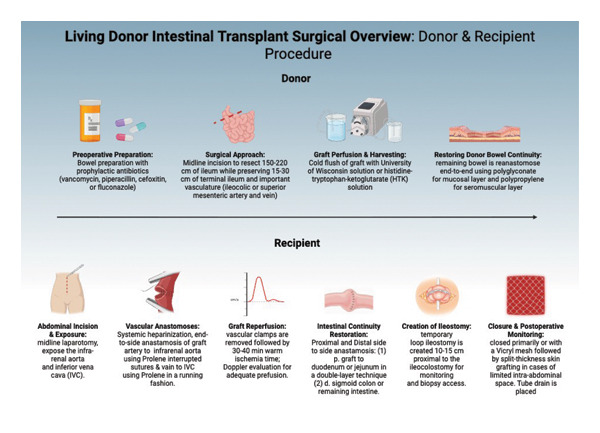
Living donor intestinal transplant surgical overview: donor and recipient procedure. Midline insertion to resect 150–220 cm of ileum while preserving 15–30 cm of terminal ileum and important vasculature for the donor, followed by proximal and distal side‐to‐side anastomosis: (1) p. graft to duodenum or jejunum in a double‐layer technique, (2) d. sigmoid colon or the remaining intestine. Created in BioRender. Basson, M. (2025) https://BioRender.com/5eo45od.

In the included studies, standard LDITx refers to the transplantation of a segmental ileal graft harvested from a living donor, with vascular anastomoses to recipient vessels and restoration of intestinal continuity. The recipient surgery begins with a midline laparotomy exposing the infrarenal aorta and the inferior vena cava to ensure proper vascular anastomoses [29, 30]. To prevent thrombosis, systemic heparinization is employed [21]. Using a continuous technique, the graft artery is sutured end‐to‐side to the aorta, and the graft vein is connected to the IVC [16, 21, 25]. Following clamp removal, the graft undergoes a period of 30–40 min of warm ischemia, during which adequate perfusion is confirmed with Doppler ultrasound [29, 32, 36]. Intestinal continuity is restored through side‐to‐side anastomoses: proximally between the graft and the duodenum or jejunum, and distally to the sigmoid colon or remaining intestine [16, 30, 37]. To facilitate postoperative monitoring, a temporary loop ileostomy is created 10–15 cm proximal to the ileocolostomy [16, 30, 33]. If the intra‐abdominal space is limited, closure may require Vicryl mesh and split‐thickness skin grafting [32, 33]. Postoperatively, a drain is placed to monitor fluid accumulation [30] (Figure 3).

#### 3.3.4. Donor Surgical Technique

To ensure donor safety and graft viability, the donor surgical process involves meticulous preparation and technique. Preoperatively, donors undergo bowel preparation alongside the administration of prophylactic antibiotics, such as vancomycin or piperacillin to reduce the risk of infection [69]. The procedure begins with a midline abdominal incision, allowing optimal access for segmental ileal resection [33, 65]. A total length of 150–220 cm of ileum is resected while preserving 15–30 cm of the terminal ileum to maintain bile acid and vitamin B12 absorption in the donor [16, 36, 69]. After resection, the graft is flushed with a cold preservation solution, either University of Wisconsin (UW) solution or histidine–tryptophan–ketoglutarate (HTK), to minimize ischemic injury [36, 65]. Bowel continuity is restored in the donor via an end‐to‐end anastomosis, while graft preparation is underway [57, 67]. The anastomosis is executed using polyglyconate sutures for the mucosal layer and polypropylene sutures for the seromuscular layer [57, 67] (Figure 3).

#### 3.3.5. Recipient Complications and Outcomes

About 1.7% of recipients experienced intraoperative complications during LDITx [29, 44, 45]. The reported incidence of postoperative ischemic necrosis is an aggregate of 2.2% [32, 40] in the total population. The aggregate incidence of pathogenic infections was 2.1% [36, 70]. Enteric leakage and discharge were observed in 0.7% of recipients [41, 53]. Vascular complications occurred in 0.7% of recipients [65]. At least one episode of graft rejection occurred in 21.2% of the total population. Long‐term nutritional independence was not sustained in 0.7% of patients [25, 60]. Postoperative ischemic necrosis is a common LDITx complication with a Clavien–Dindo (CD) classification of IIIb‐IVa. The reported incidence between the largest cohorts was 9.1% [32] and 20% [40] with an aggregate of 2.2% in the total population.

#### 3.3.6. Combined Liver and LDITx

Among the total relevant LDITx recipient population (*n* = 287), 29 recipients (10.1%) underwent transplantation that also included a liver graft. Of these, 19 recipients (6.6%) received a simultaneous living donor liver and intestinal transplant within the same operative setting. The remaining 10 recipients (3.5%) underwent combined liver–intestine transplantation in a sequential or staged manner. Reported outcomes among these recipients included episodes of acute rejection, graft loss related to ischemic or infectious complications in isolated cases, and variable mortality across cohorts. Several surviving patients achieved nutritional autonomy with the discontinuation of TPN [6, 17, 18, 26, 28, 33, 39, 40, 45, 48, 51].

### 3.4. Donor Consideration

The selection process for donors in LDITx requires thorough evaluation to ensure both graft viability and donor safety. Most donors were biologically related to the recipient, commonly parents or monozygotic twins, due to the increased HLA compatibility and availability [22, 26, 44, 48, 60, 62]. In the setting of a pediatric monozygotic twin transplant, there must be mutual agreement among the donor, their legal guardian, and the hospital’s ethics committee [59]. Proper size match between donor and recipient prevented postoperative complications, particularly those pertaining to length mismatch or volume‐related overload [16, 36, 37, 39]. ABO blood group compatibility was required to reduce the incidence of graft rejection [60]. As donor safety was a primary concern, a full preoperative assessment was conducted, including detailed clinical history, imaging studies, and psychosocial evaluations to ensure informed consent was obtained [26] (Figure 3).

### 3.5. Assessment of Reporting Quality

In this review, 11 total observational studies were evaluated by the STROBE guideline (Table 2), and 34 case reports and 9 case series were evaluated by the CARE guidelines (Table 3). Common reporting limitations were noted across the included studies. Among the observational studies, these included incomplete reporting of study design details, limited discussion of potential confounders, and inconsistent follow‐up. Among the case reports and case series, common limitations included incomplete baseline donor or recipient characterization, variable detail regarding postoperative course and long‐term follow‐up, and inconsistent reporting of complications, rejection episodes, and outcomes. These reporting limitations reduced the cross‐study comparability and should be considered when interpreting the findings.

**TABLE 2:** STROBE guideline for observational studies.

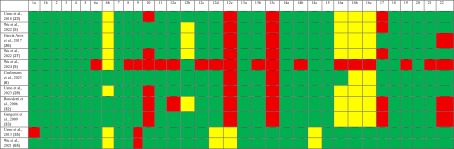

*Note:* Key: 1 for yes, 0 for no, ‐ unsure. Columns: 1: Title and Abstract—indicate the study design using a common term and provide a balanced summary of methods and findings. 2: Background/Rationale—explain the scientific background and rationale for the study. 3: Objectives—clearly state specific objectives and any prespecified hypotheses. 4: Study Design—present key elements of the design early in the paper. 5: Setting—describe the setting, locations, and relevant dates including recruitment, exposure, follow‐up, and data collection. 6a: Participants (Cohort)—give eligibility criteria, participant selection methods, and follow‐up procedures. 6a: Participants (Case–Control)—provide criteria and methods for case and control selection with rationale. 6a: Participants (Cross‐Sectional)—describe eligibility and selection methods. 6b: Matched Studies—provide matching criteria and group sizes. 7: Variables—define all outcomes, exposures, predictors, confounders, and effect modifiers, with diagnostic criteria if applicable. 8: Data Sources/Measurement—detail data sources, assessment methods, and comparability between groups. 9: Bias—describe strategies used to address potential bias. 10: Study Size—explain how study size was determined. 11: Quantitative Variables—explain handling of quantitative variables and any groupings used. 12a–e: Statistical Methods—describe all statistical methods, including for confounding, subgroups, interactions, missing data, loss to follow‐up, sampling strategy, and sensitivity analyses. 13a–c: Participants (Results)—report participant numbers at each stage, reasons for nonparticipation, and consider using a flow diagram. 14a–c: Descriptive Data—provide participant characteristics, missing data, and follow‐up time (if applicable). 15: Outcome Data—report numbers or summaries of outcome events for cohort, case–control, or cross‐sectional studies. 16a–c: Main Results—give unadjusted and adjusted estimates with precision, list confounders, and if applicable, translate relative into absolute risks. 17: Other Analyses—report additional analyses, such as subgroups or sensitivity testing. 18: Key Results—summarize findings in relation to the objectives. 19: Limitations—discuss study limitations, potential biases, and their impact. 20: Interpretation—provide a careful interpretation considering the context and supporting evidence. 21: Generalizability—discuss the external validity of findings. 22: Funding—disclose funding sources and the role of funders.

**TABLE 3:** CARE guideline for case reports.

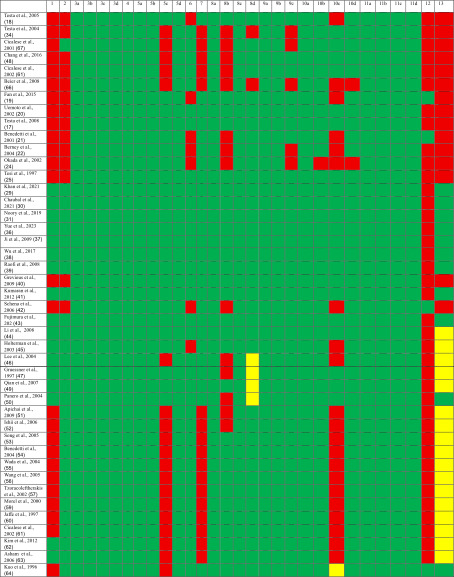

*Note:* Key: 1 for yes, 0 for no, ‐ unsure. Columns: 1: *Title*—state the primary diagnosis or intervention followed by “case report.” 2: *Key Words*—include 2 to 5 keywords that identify the diagnosis or intervention, including the term “case report.” 3a: *Abstract Introduction*—explain what makes this case unique and its contribution to the literature. 3b: *Abstract Symptoms/Findings*—summarize main symptoms and important clinical findings. 3c: *Abstract Diagnosis/Treatment/Outcome*—provide the main diagnosis, therapeutic interventions, and outcomes. 3d: *Abstract Conclusion*—offer the key takeaway from the case. 4: *Introduction*—describe why the case is unique, with optional references. 5a: *Patient Information*—present de‐identified patient‐specific details. 5b: *Primary Concerns*—list the patient’s chief symptoms and concerns. 5c: *History*—include medical, family, psychosocial, and genetic history. 5d: *Past Interventions*—note relevant prior interventions and outcomes. 6: *Clinical Findings*—report significant physical examination and clinical findings. 7: *Timeline*—organize historical and current events chronologically. 8a: *Diagnostic Testing*—detail physical examinations, laboratories, imaging, and surveys. 8b: *Diagnostic Challenges*—mention issues, such as test access, finances, or cultural barriers. 8c: *Diagnosis*—give the final diagnosis and alternatives considered. 8d: *Prognosis*—provide prognosis, including staging if applicable. 9a: *Therapeutic Interventions*—describe types (e.g., surgical and pharmacologic). 9b: *Intervention Administration*—explain dosage, strength, and duration. 9c: *Changes in Intervention*—record any changes and their rationale. 10a: *Outcomes*—include clinician‐/patient‐assessed outcomes. 10b: *Follow-Up Testing*—present follow‐up test results. 10c: *Adherence/Tolerability*—comment on adherence and tolerability, and how it was assessed. 10d: *Adverse Events*—describe any adverse or unexpected events. 11a: *Discussion Strengths/Limitations*—critically analyze strengths and limitations. 11b: *Literature Review*—discuss related literature with references. 11c: *Scientific Rationale*—support conclusions with reasoning and potential causes. 11d: *Conclusion*—provide a one‐paragraph summary of lessons learned. 12: *Patient Perspective*—include the patient’s view on their treatment experience. 13: *Informed Consent*—confirm that the patient provided informed consent.

## 4. Discussion

### 4.1. Surgical Technique Comparison of Living Donor and Cadaveric Donor

When compared to cadaveric ITx, LDITx introduced additional considerations while also potentially offering procedural advantages in select settings. Ischemia time was reduced, as the graft was immediately perfused and implanted without the delays associated with procurement and transportation required for cadaveric grafts [16, 36, 48]. The vascular anastomoses in LDITx were more controllable, and the surgical environment was typically more sterile, leading to a reduced risk of contamination [53, 67]. Ensuring donor safety and obtaining proper informed consent remained an added priority in LDITx [26, 60]. Additionally, the limited segment length that could be safely harvested from a living donor restricted the applicability of this approach for all recipients [39, 62, 67]. LDITx is a viable alternative, particularly in the setting of reduced cadaveric organ availability and accessible multidisciplinary oversight [17, 21, 34] (Figure 3).

### 4.2. Recipient Intraoperative Complication

One of the intraoperative complications was ischemia, presenting as intestinal discoloration, which occurred after reperfusion of a small bowel graft [29]. A report described venous obstruction, specifically an insufficient stoma diameter and suboptimal angle at the venous anastomosis to the inferior vena cava, causing inadequate drainage, leading to continued venous congestion [44]. Engorgement of the graft vein was observed through dark red coloration, requiring vascular reconstruction via stoma and angular enlargement [44]. In a case of graft size mismatch, a pediatric recipient’s limited intra‐abdominal space prevented abdominal cavity closure, leading to increased risk of abdominal compartment syndrome, infection, or delayed healing [45]. An absorbable mesh was used as a temporary closure, followed by a skin graft, resolving the issue with no further complications [45].

### 4.3. Recipient Postoperative Complications

#### 4.3.1. Ischemia

**FIGURE 4:**
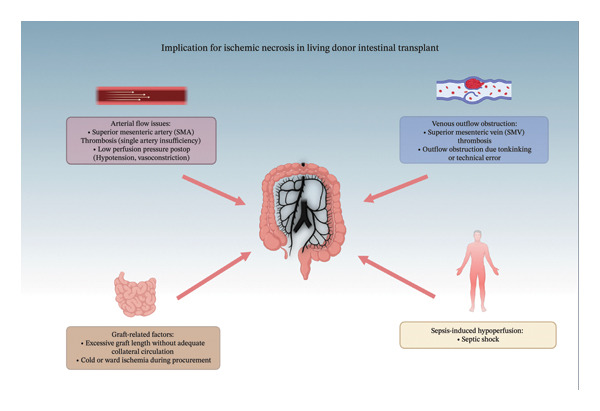
Implications for ischemic necrosis in living donor intestinal transplantation. Affected outflow, inflow, or overall general system flow contributing to ischemic necrosis. Created in BioRender. Basson, M. (2025) https://BioRender.com/b46w.gx7.

Postoperative ischemic necrosis is a common LDITx complication with a CD classification of IIIb‐IVa. Causes included septic shock [33, 40], poor arterial collateral circulation development [25], and octreotide administration [32, 67]. All patients required an immediate partial or entire resection of the intestinal allograft due to the damage, leading to continued TPN dependency and the risks associated with it [25, 32]. One method of decreasing the incidence was the insertion of donor interpositional grafts that ensured the vessels were of proper length and developed more effective collateral circulation [16] (Figure 4).

#### 4.3.2. Infection

Pathogenic infections can occur at surgical sites, due to vascular–catheter complications, or ventilation issues. These infections commonly occur within the first month post‐transplant, but can occur as far along as 90 days after [36, 70]. The most common pathogens were *Klebsiella pneumoniae*, *Staphylococcus aureus*, CMV, and EBV, with a CD classification between II and IIIb (Table 4). Multidrug‐resistant *K. pneumoniae* was observed in the allograft of one patient despite all anti‐infective regimens being employed preoperatively, leading to the eventual resection of the graft due to the pathogen being present in the donor intestinal graft before being transplanted [36]. Cytomegalovirus (CMV) and Epstein–Barr virus (EBV) infections are commonly observed post‐transplant infections due to transmission in the transplanted organ, reactivation of a latent infection because of the immunosuppressants, or after a primary infection in seronegative transplant patients. These infections can usually be treated with antiviral medications, including acyclovir, ganciclovir, and immunoglobulins, along with a temporary reduction in immunosuppressants [20, 43, 49, 77]. Viral infections can be mitigated through the use of prophylaxis, less intense immunosuppression, and shorter ischemia–reperfusion damage [16, 30]. One postoperative course was complicated by a catheter‐related infection by *S. aureus*, leading to a right jugular septic thrombophlebitis and subsequent sepsis. *S. aureus* is the most common form of bacterial infection on catheters and can be resolved through a thrombectomy of the affected vein and antibiotic administration [59, 78].

**TABLE 4:** Clavien–Dindo classification for postoperative complication.

Postoperative complication	CD class	Mitigation strategy	Level‐based evidence
Excessive bleeding from the distal colon–ileum junction	III	Postop Management: A clip placement on a hemorrhaging vessel through endoscopy [19]	6 (19)

Ischemic necrosis	IIIb‐IVa	Postop Management: Heparin can be administered to reduce the likelihood of blood clots forming in the tissue [65] Surgical Approach: Insertion of donor interpositional grafts (donor iliac artery/vein segments) can ensure the proper length of vessels and reduce the likelihood of vascular complications [16, 71]	3 (65), 3 (16), 6 (71)

Infection	II‐IIIb	Postop: Steroid pulse therapy and antiviral drugs [72] Postop: The use of antiviral prophylaxis [30] Preop: Making recipient conditions optimal [73] Periop: Less intense maintenance immunosuppression, shorter ischemia–reperfusion damage, and reduced ACR episodes account for decreased incidence of viral infection [16]	6 (72), 6 (30), 1 (73), 3 (16)

Anemia	II	Postop: Transfusion of donor‐specific RBCs, increased immunosuppression, and plasmapheresis [74]	6 (74)

Pelvic hematoma	IIIb	Use of an acellular dermic matrix to properly close the abdomen and prevent it from excessively retracting [63]	6 (63)

Metabolic acidosis	II	Postop: Intensive supportive care until symptoms subside [75]	1 (75)

Vascular thrombosis	IV‐V	Surgical approach, instead of directly anastomosing the graft, takes parts of the donor SMA and SMV to elongate the vessels and allow easier connection to the surrounding vasculature [65]	3 (65)

Enteric leakage	II	Postop: Revert back to TPN from enteral feeding [41] Postop: Increase in immunosuppressive drug dosage to alleviate immune response to areas of ischemic injury and allow proper healing of graft [76]	6 (41), 1 (76)

*Note:* Key: The Clavien–Dindo classification system categorizes postoperative complications by severity. Grade I includes minor deviations not requiring specific treatment. Grade II involves complications requiring medications, such as antibiotics or blood transfusions. Grade III requires surgical, endoscopic, or radiological intervention, with IIIa performed without general anesthesia and IIIb with it. Grade IV refers to life‐threatening complications needing intensive care, with IVa involving single‐organ dysfunction and IVb involving multiorgan failure. Grade V indicates patient death. Key: Levels of evidence indicate the methodological strength of studies. Level 1 includes systematic reviews and meta‐analyses, which combine results from many studies for the most reliable conclusions. Level 2 is randomized controlled trials that reduce bias by randomly assigning participants to groups. Level 3 includes cohort studies that follow people over time to see how exposures affect outcomes. Level 4 is case–control studies that compare people with and without a condition, often used for rare diseases. Level 5 includes cross‐sectional studies that look at data at a single point in time, showing associations but not cause and effect. Level 6 covers case reports and series, which describe individual cases but lack generalizability. Level 7 is expert opinion, based on experience rather than research, and is the least reliable.

#### 4.3.3. Enteric Leakage

An instance of enteric leakage was observed 23 days postop, occurring in a location of the graft that was neither on the surface nor near the direct anastomoses, with no conclusive etiology [41]. The course of treatment was conservative, consisting of TPN re‐administration, which ultimately reduced the volume of the leakage [41]. Another recipient experienced increasing intestinal fistula discharge a month after the procedure, shortly after reducing immunosuppression, which indicated acute rejection [53]. This can be attributed to intestinal transplants being hypersensitive to cold ischemia, with ischemic injury occurring regardless of administered precautions, which disrupts the intestinal mucosal barrier [76]. This increases the wall permeability, leading to intestinal flora penetration along with intestinal fluid exudation, and in reduced immunosuppression settings, the immune system triggers subsequent inflammation, sepsis, and organ dysfunction [76]. A large dosage of immunosuppressive agents was employed, which resolved the rejection shortly after, leading the fistula to completely heal and the patient to make a full recovery [53]. The CD classification for this complication is II due to the utilization of pharmaceutical intervention instead of surgery (Table 4).

#### 4.3.4. Vascular Complications

Vascular complications included vascular thrombosis, arterial stenosis, and pseudoaneurysms [65]. The appropriate CD classification is IV‐V (Table 4). Increased vascular rupture in the event of pancreatic leakage is attributed to direct vascular anastomoses being found behind the pancreas [65]. Prevention includes the utilization of vascular reconstruction of the donor terminal superior mesenteric artery (SMA) and superior mesenteric vein (SMV) with autologous internal iliac vessels to elongate the vessels to allow easier connection to the recipient’s inferior aorta, vena cava, and superior mesenteric vessels [65]. A recipient experienced inferior epigastric artery erosion 30 days post‐transplant, leading to a massive intra‐abdominal bleed, which required packing and suture ligation [41]. When vascular complications arise, it is necessary to perform immediate surgery to preserve both the life of the recipient and as much of the graft as possible.

#### 4.3.5. Miscellaneous

Rare occurrences of severe hemolytic anemia caused by “passenger” lymphocytes from the donor can cause passenger lymphocyte syndrome (PLS) in the recipient [50]. Without treatment, it can result in hypotension, acute renal failure, intravascular coagulation, and subsequent multiorgan failure [79]. This can be treated through transfusion of donor‐specific RBCs, increased immunosuppression, and plasmapheresis, corresponding to a CD classification of II (Table 4) [74].

There was a single occurrence of pelvic hematoma occurring due to graft edema combined with a contracted abdominal cavity. This causes a defect in fascia closure, leading to the occurrence of a hematoma. This can be treated through surgical intervention and the use of an acellular dermal matrix to properly close the abdomen, which gives it a IIIb rating on the CD classification (Table 4) [63].

Metabolic acidosis was another possible complication related to the increased bicarbonate content of the GI tract [64]. After a procedure, a diarrheal state may follow, which results in the excretion of too many bicarbonate ions, leading to an ionic imbalance [75]. This can be treated with intense supportive care, making it a CD II (Table 4).

### 4.4. Donor Postoperative Complications

All donors had an uneventful recovery from segmental resection of their bowels. Although a common contraindication to performing LDITx was the potential risks to the donors, most adverse effects of the procedure were acute, mild diarrhea and malabsorption that resolved on their own in most cases [6, 16, 20, 21, 25–29, 32, 37, 47, 49]. No donor deaths were reported in any of the included studies. The CD classification given to this condition is between I and II.

### 4.5. Outcomes

#### 4.5.1. Graft Survival and Rejection Rate

Rejection episodes are typically identified through ileoscopic biopsy, ileostomy output changes, or histological findings, such as crypt apoptosis and mucosal lymphocytic infiltration [80]. A rejection may be recurrent in settings of reduced immunosuppression or poor compliance [16]. Rejection is a notable issue when it comes to managing LDITx recipients due to the strong immunogenic nature of the grafts. Although the incidence of acute rejection is common, factors, such as suboptimal HLA compatibility, subtherapeutic dosing levels for immunosuppressive medications, and serostatus mismatch for CMV or EBV, dictate its severity [16, 81].

The presence of pre‐formed donor‐specific antibodies (DSAs) and immunosuppression due to infection or toxicity are contributors to both cellular and antibody‐mediated rejection (ABMR). Inadequate nutrition status and the absence of formal surveillance protocols delay the identification of rejection, leading to increased graft damage [65, 82]. One effective method in the prevention of rejection is a triple immunosuppressive drug regimen of tacrolimus, mycophenolate mofetil (MMF), and corticosteroids [16]. Perioperatively, the use of induction therapy with lymphocyte‐depleting agents can reduce T‐cell activation and subsequent rejection [82]. Emerging noninvasive biomarkers, such as donor‐derived cell‐free DNA and fecal inflammatory markers, are now being evaluated as adjuncts to histologic monitoring [83, 84]. Mild to moderate acute cellular rejection (ACR) is treated with high‐dose intravenous corticosteroids. Steroid‐resistant patients may require the use of agents, such as ATG or switching to alternative immunosuppressants. For ABMR, multimodal therapy involving plasmapheresis, IVIG, and rituximab is employed [38]. In chronic rejection, clinicians may recheck drug levels, screen for infection, or adjust maintenance regimens to sustain graft viability. Though not all rejection episodes are followed by graft failure, frequent or severe episodes are associated with an increased risk for chronic rejection and loss of nutritional autonomy [16, 83].

A cohort of 54 patients underwent LDITx, with 25.9% experiencing at least one episode of acute rejection, and 5.6% developing chronic rejection [5]. This study also reported that recipients with greater HLA compatibility (> 3 matches) had significantly better patient and graft survival outcomes, suggesting an immunological advantage unique to living donor selection [16].

#### 4.5.2. Quality of Life and Nutritional Autonomy

The primary goal of LDITx is to restore enteral feeding and prevent dependence on TPN. Patients who were completely weaned off TPN showed dramatic signs of recovery, including substantial weight gain, catch‐up growth to age‐related percentiles, and successful transition to enteral or oral nutrition [17, 21, 24, 29, 31, 37–40]. Retransplantation occurred in 1% of patients [16, 20]. Outcomes of LDITx appear to be influenced by the severity of underlying disease, surgical complications, and graft function over time.

#### 4.5.3. Mortality

##### 4.5.3.1. Infection‐Related Mortality

Infectious complications appear to represent the leading cause of mortality following LDITx, frequently occurring in the setting of intensive immunosuppression and, in some cases, concomitant rejection, which allowed opportunistic infections, such as *Pneumocystis carinii* and *Aspergillus* to occur, both associated with mortality at 16 months and 3 months post‐transplant, respectively [20, 32]. Sepsis was a major contributor to infectious deaths arising in different clinical settings, such as postoperative complications including wound dehiscence with enterocutaneous fistula and excessive immunosuppression, which led to the deaths of recipients 43 days and up to several months post‐transplant, respectively [41, 44]. One recipient also developed ACR and succumbed to complicated sepsis 77 months later [48]. Additionally, infection contributed to mortality in recipients experiencing rejection, showcasing the compounded risk associated with immunologic instability. Overall, infection plays a crucial role as a driver for both early and late mortality in LDITx recipients.

##### 4.5.3.2. Rejection‐Related Mortality

Deaths due to rejection were less commonly reported but remain a crucial contributor to mortality. Rejection was implicated in 2 deaths, comprising a chronic rejection death and an acute rejection death. A toddler died 16 months post‐transplant due to chronic rejection in conjunction with an infectious complication [20]. One recipient developed ACR complicated with sepsis, which resulted in death 77 months later [48]. These findings suggest that while rejection alone may be less commonly reported as a direct cause of death, it plays a significant role in predisposing patients to secondary complications, particularly infection.

##### 4.5.3.3. Post‐Transplant Lymphoproliferative Disease (PTLD)

PTLD was also an uncommon cause of death that presented as a severe condition. Among reported cases, one recipient died 5 months post‐transplant due to PTLD‐associated multisystem organ failure, representing one of four patients affected by this complication [26]. An effective treatment method involves using rituximab alone or in combination with methylprednisolone and cyclophosphamide, but failure could necessitate graft enterectomy [26]. Although a rare occurrence, PTLD carries substantial mortality risk and reflects the oncogenic potential of prolonged immunosuppression in this LDITx population.

#### 4.5.4. Length of Stay

Patients’ length of stay ranged from 11 to 293 days. Duration of stay is chiefly correlated with TPN dependence. In several studies, the patient went home after tolerating full enteral feeding (Supporting Table 2).

#### 4.5.5. Comparison Between Living and Cadaveric Donor ITx

While LDITx is often indicated for cases involving time sensitivity and donor compatibility constraints [16, 27, 30], a comparison with its cadaveric counterpart should be investigated. Due to ethical and logistical limitations, a randomized controlled trial comparing the two directly is highly unlikely; however, there are three retrospective observational studies [6, 16, 35]; in this systematic review, it offers an attempted head‐to‐head comparison.

Evidence suggests no statistically significant difference between the two procedures in terms of patient and graft survival, rejection rates, and postoperative complications [6]. This retrospective study suggested that the two procedures are broadly comparable in long‐term outcomes when appropriately matched based on their analysis of 3965 deceased donor intestinal transplant (DDITx) cases and 76 LDITx cases. Due to the considerable disparity in size between the two cohorts, generalizability is slightly limited in this case.

While some retrospective observational studies have suggested potential advantages of LDITx over DDITx in select clinical settings, Wu et al. found LDITx to have significantly higher patient survival at 1 and 3 years and a lower incidence of acute rejection [16]. Ueno et al. similarly observed significantly higher survival rates at 1 and 5 years for its pediatric recipients, as well as significantly fewer infectious complications when compared to cadaveric donors [35]. None of the three studies referenced quality of life directly, but Ueno et al. and Wu et al. noted achieving nutritional autonomy sooner in LDITx recipients, although the significance of this comparison was unmeasured. They also mentioned potential advantages, such as elective operative planning and minimized ischemia time in LDITx recipients [16, 35].

Given the limitations of existing comparisons and the rarity of LDITx, a well‐powered multicenter retrospective analysis with standardized outcomes and sophisticated multivariate analysis is needed to further clarify the comparison between a living donor and a cadaveric donor.

### 4.6. Limitation

This systematic review may have limitations due to the data being collected retrospectively, which in turn could have introduced study selection bias and misinterpretation of the results. Another potential limitation is human error, especially given the large volume of studies that were screened and analyzed. Additionally, the variation in the quality of the data and the completeness of the data could have impacted the overall robustness of the review due to inconsistencies. There was significant heterogeneity among the included studies (observational studies, case series, and case reports) that differed in methodology, sample size, and completeness of reporting. Case reports and small case series often describe unique or successful clinical experiences and may therefore be subject to reporting and publication bias. In addition, these study designs typically involve small sample sizes and lack standardized outcome reporting. As a result, combining evidence across these heterogeneous sources may limit the generalizability of the findings. Furthermore, because the included studies were mainly observational, this can lead to causal inference. Additionally, follow‐up duration was inconsistently reported across the included studies, and several studies did not specify follow‐up periods, which limits the ability to fully evaluate long‐term morbidity and mortality after transplantation.

## 5. Conclusion

LDITx is a valuable alternative to its cadaveric counterpart, avoiding issues related to time constraints and donor availability. The procedure has risks of postoperative complications, such as infection and ischemia, but effective strategies exist to prevent or lessen their impact. Additionally, close monitoring and optimization of immunosuppression can help reduce rejection episodes. Successful transplantation has enabled many patients to achieve TPN independence and an improved quality of life. Although direct comparisons are limited, existing data suggest that LDITx is comparable to DDITx and may offer certain advantages, including fewer infectious complications, faster nutritional autonomy, higher patient survival, more flexible scheduling of elective surgeries, and reduced ischemia time. A large, comprehensive multicenter retrospective study with standardized outcomes and multivariate analysis is needed to verify these benefits. Clinically, LDITx should be considered for patients with chronic IF who develop life‐threatening TPN‐related complications or have access to a suitable living donor, as it may facilitate earlier transplantation and minimize prolonged exposure to TPN‐related health issues. Overall, LDITx is an effective option for patients with chronic IF experiencing severe TPN complications, though further research is necessary to guide medical decisions for both healthcare providers and patients.

## Funding

The authors have nothing to report.

## Conflicts of Interest

The authors declare no conflicts of interest.

## Supporting Information

Additional supporting information can be found online in the Supporting Information section.

## Supporting information


**Supporting Information** Supporting Table 1: Search strategy. Supporting Table 2: Summary of outcomes. Supporting Table 3: Risk of bias (ROBINS‐I) for observational studies.

## Data Availability

Data sharing is not applicable, as no new data were collected for this study. All analyses and aggregated calculations were based on previously published data, which are cited within the manuscript.
