# Supplementary material for: Living Donor Intestinal Transplant: Indication, Techniques, Surgical Complications, and Outcomes in Recipients and Donors: A Systematic Review
Source: J Transplant. 2026 May 31;2026:4030621. doi: 10.1155/joot/4030621 (PMC13239233; doi:10.1155/joot/4030621)
Supplement: Supplementary file 1 — Supporting Information Supporting Table 1: Search strategy. Supporting Table 2: Summary of outcomes. Supporting Table 3: Risk of bias (ROBINS‐I) for observational studies. [file JOOT-2026-4030621-s001.docx]

**Supplementary Table 1: Search Strategy**

|  | - PubMed - Cochrane - Web of Science - VHL | (living donor intestine transplant OR living donor intestinal transplant OR living donor intestine transplantation OR living donor intestinal transplantation OR Living Donor Bowel Transplant OR Living Donor Bowel Transplantation OR Living Intestinal Transplant Surgery OR Living Intestine Transplant Surgery OR Intestinal Transplantation from Living Donor OR Intestine Transplantation from Living Donor OR Bowel Transplantation from Living Donor OR Living Donor Digestive Tract Transplant) AND (Postoperative Complications OR "Postoperative Complications"[Mesh] OR complications OR Intraoperative complications OR "Intraoperative Complications"[Mesh] OR Patient Outcome Assessment OR "Patient Outcome Assessment"[Mesh] OR Indication OR "Contraindications"[Mesh] OR Contraindications)  (living donor intestine transplant OR living donor intestinal transplant OR living donor intestine transplantation OR living donor intestinal transplantation OR Living Donor Bowel Transplant OR Living Donor Bowel Transplantation OR Living Intestinal Transplant Surgery OR Living Intestine Transplant Surgery OR Intestinal Transplantation from Living Donor OR Intestine Transplantation from Living Donor OR Bowel Transplantation from Living Donor OR Living Donor Digestive Tract Transplant) AND (Postoperative Complications OR Post-surgical Complications OR Surgical Morbidity OR Postoperative Adverse Events OR Post-surgical Issues OR Surgical Recovery Complications OR Surgical Outcomes OR Post-surgical Morbidity OR Postoperative Events OR Complications Following Surgery OR Complications OR Adverse Events OR Health Issues OR Medical Complications OR Surgical Complications OR Procedure-Related Issues OR Post-treatment Complications OR Clinical Complications OR Treatment-Related Complications OR Intraoperative Complications OR Surgical Procedure Complications OR Intraoperative Adverse Events OR In-Surgery Complications OR Operative Complications OR Surgical Technique Complications OR Intraoperative Morbidity OR Complications During Surgery OR Surgical Process Complications OR Patient Outcome Assessment OR Clinical Outcome Assessment OR Surgical Outcome Assessment OR Patient Prognosis OR Health Outcome Evaluation OR Patient Treatment Outcomes OR Therapeutic Outcome Assessment OR Post-treatment Evaluation OR Patient Recovery Outcomes OR Patient Health Metrics OR Outcome Indicators OR Indication OR Treatment Indication OR Therapeutic Indications OR Medical Indications OR Clinical Indication OR Reason for Treatment OR Justification for Procedure OR Indication for Surgery OR Diagnostic Indications OR Contraindications OR Treatment Contraindications OR Therapeutic Restrictions OR Clinical Contraindications OR Surgical Contraindications OR Procedure Limitations OR Adverse Treatment Indicators OR Contraindication for Surgery OR Risk Factors for Treatment OR Contraindicating Conditions)  (ALL=(living donor intestine transplant OR living donor intestinal transplant OR living donor intestine transplantation OR living donor intestinal transplantation OR Living Donor Bowel Transplant )) AND ALL=(Postoperative Complications OR Post-surgical Complications OR complications OR Intraoperative complications OR Surgical Procedure Complications OR Intraoperative Adverse Events OR Patient Outcome Assessment OR Surgical Outcome Assessment OR Outcomes OR Indication OR Contraindications)  ("living donor intestine transplant" OR "living donor intestinal transplant" OR "living donor intestine transplantation" OR "living donor intestinal transplantation" OR "Living Donor Bowel Transplant" OR "Living Donor Bowel Transplantation" OR "Living Intestinal Transplant Surgery" OR "Living Intestine Transplant Surgery" OR "Intestinal Transplantation from Living Donor" OR "Intestine Transplantation from Living Donor" OR "Bowel Transplantation from Living Donor" OR "Living Donor Digestive Tract Transplant") AND ("Postoperative Complications" OR "Post-surgical Complications" OR "Surgical Morbidity" OR "Postoperative Adverse Events" OR "Post-surgical Issues" OR "Surgical Recovery Complications" OR "Surgical Outcomes" OR "Post-surgical Morbidity" OR "Postoperative Events" OR "Complications Following Surgery" OR Complications OR "Adverse Events" OR "Health Issues" OR "Medical Complications" OR "Surgical Complications" OR "Procedure-Related Issues" OR "Post-treatment Complications" OR "Clinical Complications" OR "Treatment-Related Complications" OR "Intraoperative Complications" OR "Surgical Procedure Complications" OR "Intraoperative Adverse Events" OR "In-Surgery Complications" OR "Operative Complications" OR "Surgical Technique Complications" OR "Intraoperative Morbidity" OR "Complications During Surgery" OR "Surgical Process Complications" OR "Patient Outcome Assessment" OR "Clinical Outcome Assessment" OR "Surgical Outcome Assessment" OR "Patient Prognosis" OR "Health Outcome Evaluation" OR "Patient Treatment Outcomes" OR "Therapeutic Outcome Assessment" OR "Post-treatment Evaluation" OR "Patient Recovery Outcomes" OR "Patient Health Metrics" OR "Outcome Indicators" OR Indication OR "Treatment Indication" OR "Therapeutic Indications" OR "Medical Indications" OR "Clinical Indication" OR "Reason for Treatment" OR "Justification for Procedure" OR "Indication for Surgery" OR "Diagnostic Indications" OR Contraindications OR "Treatment Contraindications" OR "Therapeutic Restrictions" OR "Clinical Contraindications" OR "Surgical Contraindications" OR "Procedure Limitations" OR "Adverse Treatment Indicators" OR "Contraindication for Surgery" OR "Risk Factors for Treatment" OR "Contraindicating Conditions") |
| --- | --- | --- |
| Date initiated | 12/24/2024 | |
| Initial findings | Total= 1039   - PubMed: 562 - Cochrane: 96 - VHL: 12 - WOS: 369 | |
| Removed before  screening (Duplicates) | Excluded = 205  Included = 834 | |

**Supplementary Table 2: Summary of Outcomes**

| **Author, Year** | **Condition** | **Indication** | **Recipient Technique** | **Recipient Postoperative Complications & Incidence** | **Donor Technique** | **Donor Postoperative Complications & Incidence** | **Patient Rejection Incidence** | **Number of rejection episodes per patient** | **Recipient Mortality & Incidence** | **Recipient Length of stay** | **Other outcomes** | **Risk factors** |
| --- | --- | --- | --- | --- | --- | --- | --- | --- | --- | --- | --- | --- |
| Testa *et al.*, 2008 | Gastroschisis (2), Midgut volvulus + megacystic microcolon (2) | Compatible donor (1) and poor clinical conditions in (2) | Staged liver-intestine transplantation | None | - | - | - | - | None | 115-293 days postop | At final follow up all children had gained weight and height, with 3 catching up to the average | - |
| Testa et al., 2005 | gastroschisis and intestinal malrotation, and lost all small bowel and colon | Mother was a compatible donor | staged liver-intestine transplantation with a piggyback liver transplantation technique, followed by an isolated intestinal transplantation | severe steatohepatitis | Segmental Ileal Resection with Ileocolic Vascular Pedicle | None | None | None | - | - | Ileostomy taken down after a month without any complications | Young age, high-performing reactive antibody panel |
| Wu et al., 2024 | - | - | Isolated LDITx | - | - | - | - | - | 13 | - | Diarrhea, weight loss, and vitamin B12 deficiency were common following surgery, and disappeared in most cases within a year | - |
| Fan et al., 2015 | sudden onset of severe bowel infarction | Not enough time to find a cadaveric donor | isolated intestinal transplantation with splenectomy | Excessive bleeding from the distal colon and ileum connections and an intrabdominal abscess | Segmental Ileal Resection with Ileocolic Vascular Pedicle | Acute mild diarrhea | 1 | 1 | - | - | - | - |
| Uemoto et al., 2002 | All had short bowel syndrome | All had a compatible mother donor | isolated LDITx | - |  |  | 3 | 4, 4, 1 | 1 | 14-16 days | One patient lost the transplant and was retransplanted with the cadaveric donor, and another is off TPN and doing well | - |
| Benedetti et al., 2001 | massive bowel necrosis | Sister was a compatible donor | isolated LDITx | None | Segmental Ileal Resection with Ileocolic Vascular Pedicle | Acute mild diarrhea | None | None | None | 10 days | D-xylose 50% normal value at 1 year postop, returned to preinjury weight, and was able to maintain it and live a normal life | - |
| Berney et al., 2004 | short gut syndrome | Compatible identical twins and time sensitivity | isolated LDITx | None | Segmental Ileal Resection | None | - | - | - | 53 days | - | Significant weight deficit |
| Ueno et al., 2018 | Short gut syndrome (9), motility disorders (15), retransplant cases (3) | Short gut syndrome (9), motility disorders (15), retransplant cases (3) | - | - | - | - | - | - | - | - | No significant difference from cadaveric donors | - |
| Okada et al., 2002 | Microvillus inclusion disease | Grandmother was a compatible donor | isolated LDITx | - |  |  | - | - | None | - | Completely off TPN, and is almost normal | - |
| Tesi et al., 1997 | Gardner's syndrome (1), intestinal dysmobility + pseudo-obstruction (1) | - | isolated LDITx | Loss of distal 20 cm of the ileum graft due to poor development of arterial collateral circulation (1) | Segmental jejunal intestinal allograft harvesting | slight malabsorption (1) | 2 | 1, 2 | None | 60 days (1), 30 days (1) | Both returned to TPN | A large number of small arteries and veins on the allograft |
| Wu et al., 2022 | - | time-sensitivity, compatible donor | isolated LDITx | - | Segmental small bowel resection technique | Acute mild diarrhea | 14 | 8 | 12 | - | posttransplant lymphoproliferative disease (1), no graft-versus-host disease, one graft over 23 years working fine, all off TPN | - |
| Garcia Aroz et al., 2017 | Either gastrochisis (6), megacystis microcolon (1), Midgut volvulus (1), NEC (1), SMA thrombosis (1) | Cholestatic liver disease (3), liver failure (5), loss of central vein access (2) | Combined living intestinal-liver transplant (2), isolated LDITx (1) | 7 out of 13 | Segmental Ileal Resection with Ileocolic Vascular Pedicle | Acute mild diarrhea | 2 | 2 | 3 | - | - | - |
| Wu et al., 2022 | Intestinal failure | time-sensitivity | - | - | Segmental Ileal Resection with Ileocolic Vascular Pedicle | Small bowel adhesions, acute mild diarrhea | 8 | 8 | 12 | - | - | - |
| Ceulemans et al., 2023 | - | - | - | - | Living-Related Segmental Ileal Resection | Acute mild diarrhea | 47% | - | 17 (1 yr)/ 31 (5 yr)/ 34 (10 yr) | - | - | - |
| Ueno et al., 2023 | Intestinal failure due to different causes | Intestinal failure | - | 7 (53.8%) | Living Donor Ileal Resection | Acute mild diarrhea | - | - | - | - | survival rates were comparable to cadaveric donors | - |
| Khan et al., 2021 | short bowel syndrome | The daughter was a compatible donor | Intestinal transplant (ITx) with vascular anastomosis revision | - | Living Donor Small Bowel Resection | Acute mild diarrhea | - | - | None | - | The patient is thriving on an enteral diet | - |
| Chaubal et al., 2021 | short bowel syndrome | time sensitivity, the father was a compatible donor | isolated LDITx | - | Living Donor Ileal Resection | None | None | None | None | 36 days | Sepsis-free without recurrence of COVID-19 symptoms | COVID-19 |
| Noory et al., 2019 | short bowel syndrome | time-sensitivity, the sister was a compatible donor | isolated LDITx | - | Living Donor Ileal Resection | None | - | - | None | - | Able to get off TPN, gain weight, but developed anal cancer later | - |
| Benedetti et al., 2006 | Irreversible intestinal failure | time-sensitivity, compatible donor | isolated LDITx | none | Living Donor Ileal Resection | Acute mild diarrhea | 3 | 1,1,1 | 1 | 13-290 days | All pediatric patients had at least 1 central line sepsis post-transplant, all living recipients gained weight and height, and everyone was off TPN | - |
| Gangemi et al., 2009 | Intestinal failure | Intestinal failure | combined living donor intestinal and liver transplant (5), isolated LDITx (5) | 1 loss to ischemic necrosis, another to posttransplant lymphoproliferative disease | Living Donor Ileal Resection | None | 2 | 2, 1 | 3 | - | 6/7 living are off TPN, isolated LDITx turned out worse than combined | Pediatric patients with a weight under 8 kg |
| Testa et al., 2004 | Irreversible intestinal failure | Irreversible intestinal failure | - | - | Living Donor Ileal Resection | None | - | - | 3 | - | All off TPN | - |
| Ueno et al., 2013 | Short bowel syndrome (7), Intestinal functional disorder (2), Retransplant (1) | intestinal failure | - | - | - | - | - | - | 4 | - | Lower graft survival rate than cadaveric donors | high rejection rates |
| Yue et al., 2023 | short bowel syndrome | Father was a compatible donor | isolated LDITx | Multidrug-resistant K pneumoniae infection | Living Donor Ileal Resection | - | 1 | - | - | - | Graft was lost | - |
| Ji et al., 2009 | short bowel syndrome | time-sensitivity, compatible donor | isolated LDITx | heart and lung failure (1) | Living Donor Ileal Resection | Acute mild diarrhea and bloating | 3 | 1, 2, 3 | 1 | - | All off TPN | - |
| Wu et al., 2017 | short bowel syndrome | Compatible donor | isolated LDITx | None | Living Donor Ileal Resection | None | 1 | 1 | None | - | Functional transplant after 17 years, and off TPN | - |
| Raofi et al., 2008 | Megacystis-microcolon-intestinal hypoperistalsis syndrome | time-sensitivity, the mother was a compatible donor | Multivisceral transplantation | - | Living Donor Ileal Resection | - | None | None | None | - | Normal absorption limits, the height and weight had caught up to average and above average | - |
| Grevious et al., 2009 | Irreversible intestinal failure | Mothers were compatible donors | combined living donor intestinal and liver transplant | 1 graft loss to ischemic sepsis | Living Donor Ileal Resection | None | None | None | None | - | TPN was discontinued in all | - |
| Kumaran et al., 2012 | short bowel syndrome | shortage of cadaveric donors, Son was a compatible donor | Isolated LDITx | Enteric leakage (misc.) | Living Donor Ileal Resection | None | 1 | 1 | 1 | 43 days (till death) | - | malabsorption and an increased frequency of defecation |
| Schena et al., 2006 | Churg-Strauss syndrome, ultra-short bowel syndrome | Identical twin, time-sensitivity | Isolated LDITx | None | Living Donor Ileal Resection | None | None | None | None | - | Doing well 27 months later | asthma |
| Fujimura et al., 2021 | hypogenic type of intestinal dysganglionosis | Hypogenic type of intestinal dysganglionosis, the father was a compatible donor | Isolated LDITx | Two episodes of steroid-responsive mild ACR and cytomegalovirus (CMV) infection | Living Donor Ileal Resection | - | None | None | None | - | Patient is stable 5 years later | - |
| Li et al., 2008 | Short gut syndrome; small bowel resection (necrosis due to volvulus); necrosis due to obstruction | Parent Available | Isolated Living Donor Intestinal Transplant (LDITx) using Distal Ileum Grafts | None | Living Donor Ileal Resection | - | 2 | 1,1 | Fatal sepsis (at 5 months), 1 | 40 days | Adjustment of immunosuppressors is crucial for the recipients to experience high-quality lives. | - |
| Cicalese et al., 2001 | Short bowel syndrome due to trauma, superior mesenteric artery thrombosis | ABO compatible donor | Isolated Living Donor Intestinal Transplant (LDITx) using a Segment of Ileum Graft | None | Living Donor Ileal Resection | - | 1 | 1 | - | - | All donors had an uneventful recovery | - |
| Holterman et al., 2003 | Profound malnutrition (SBS), advanced TPN-induced liver failure, born with gastroschisis | Parent Available, time constraint on available cadaveric bowel | Isolated Living Donor Intestinal Transplant (LDITx) using an Ileocolic Pedicle Graft | EBV infection | Living Donor Ileal Resection | - | None | none | - | - | - | cholestatic jaundice, Gastrochisis defect |
| Lee et al., 2004 | Short bowel surgery (jejunocolostomy due to mesenteric vein thrombosis), 30 cm jejunum and colon (distal to splenic flexure), partially unbalanced renal function, 2+ years of home TPN, occluded superior vena cava and subclavian veins, inferior VC line remained | Daughter donor, repeated life-threatening infection | Isolated Living Donor Intestinal Transplant (LDITx) using a Jejuno-Ileal Graft. | None | Segmental Small Bowel Resection | - | - | - | - | 42 days | Day 70, the MMF was ceased | - |
| Gruessner et al., 1997 | Paraplegic, life-threatening TPN complications (lack of vascular access, recurrent infections, intermittent liver dysfunction), severe motor vehicle accident, superior mesenteric artery injury, bowel resection (25 cm small bowel, sigmoid colon, rectum, proximal jejunostomy) | Parent Available, Weight-matched cadaver donor unavailable | Isolated Living Donor Intestinal Transplant (LDITx) with an Ileocolic Segment Graft. | None | Segmental Ileum Resection with Ileocolic Vascular Pedicle | Mild loose stools | none | none | - | 21 days | completely off TPN since discharge (posttransplant day 21), has gained 20 kg, | - |
| Chang et al., 2016 | Hirschsprung disease, trauma, mesenteric vessel thrombosis | Family available | Isolated Living Donor Intestinal Transplant (LDITx) with End-to-End Microsurgical Anastomosis. | - | Segmental Small Bowel Resection | - | 1 | 1 | After many years, Acute cellular rejection led to death (2-year-old),1 | - | kidney Tx from the same living donor | Hirschsprung Disease, Trauma, Mesenteric vessel thrombosis |
| Qian et al., 2007 | Short bowel syndrome, volvulus necrosis, subtotal enterectomy | Potential advantages of LR | Living-Related Segmental Bowel Transplantation (LR-SBT) | Postoperative cytomegalovirus infection | Segmental Ileal Resection with Ileocolic Vascular Pedicle | Loose stools | 1 | 1 | - | - | The recipient is alive at 15 months with 8 kg an increase in weight. | - |
| Panaro et al., 2004 | Short bowel syndrome (gastroschisis), 150-cm ileal graft (blood group O mother), multiple line sepsis episodes, progressive cholestasis (bilirubin 15 mg/dL), weight 10.8 kg, shrunken abdominal cavity (22 laparotomies), required periodic blood transfusions. | Parent Available | Living-Related Isolated Ileum Transplantation (LRIIT) | life-threatening hemolytic anemia | Segmental Ileal Resection with Ileocolic Vascular Pedicle | none | - | none | - | - | - | - |
| Apichai et al., 2009 | Gastroschisis is complicated by intestinal volvulus, chronic liver failure (TPN-induced cholestatic liver disease), and multiple infections (including peritonitis). | Parent Available | Living-Donor Combined Small Bowel and Liver Transplantation | None | - | none | - | none | - | - | Five months post-transplant, she developed sudden gastrointestinal bleeding | - |
| Ishii et al., 2006 | Short bowel syndrome, TPN-dependent (male, hypoganglionosis), massive enterectomy (female, due to volvulus). | Parent Available | Isolated Living Donor Intestinal Transplant (LDITx) with Ileal Graft and End-to-Side Anastomosis | None | Harvesting up to one-third of the distal ileum | none | 2 | 1,1 | - | 112 days | - | - |
| Song et al., 2005 | Short Gut syndrome | Parent Available | Isolated Living Donor Intestinal Transplant (LDITx) with End-to-Side Arterial Anastomosis | Increased discharge at the intestinal fistula site, causing rejection | Segmental small bowel resection with vascular anastomosis | none | 1 | 1 | - | - | - | - |
| Benedetti et al., 2004 | Blunt trauma, GSW | Family available, risks associated with TPN | Isolated Living Donor Intestinal Transplant (LDITx) | None | Gruessner and Sharp's technique for segmental bowel graft procurement | None | 1 | 1 | - | - | - | - |
| Wada et al., 2005 | Short bowel syndrome, TPN-dependent (hypoganglionosis, male), massive enterectomy (due to volvulus, female). | Parent Available | Isolated Living Donor Intestinal Transplant (LDITx) | None | Segmental bowel resection with vascular anastomosis | none | 2 | 1,1 | - | 112 days | - | - |
| Wang et al., 2005 | Acute abdominal pain, massive, small intestine, and ascending colon resection (necrotic volvulus) | Parent Available | Isolated Living Donor Intestinal Transplant (LDITx) | - | Gruessner and Sharp's technique for living-related small bowel transplantation | none | none | none | - | - | - | - |
| Tzoracoleftherakis et al., 2002 | Multiple episodes of line sepsis (complication of initial injury), incipient cholestasis (related to TPN, bilirubin 3.2 mg/dL), no cirrhosis (liver biopsy). | Parent Available | Isolated Living Donor Intestinal Transplant (LDITx) | - | Gruessner and Sharp's technique for living-related small bowel transplantation. | none | none | none | - | - | - | - |
| Cicalese et al., 2002 | - | Associated risk of cadaveric bowel | Living Related Small Bowel Transplantation (LR SBTx) with HLA Matching and Immunosuppressive Protocol | None | - | - | none | none | - | - | - | - |
| Morel et al., 2000 | Short gut syndrome, extensive intestinal resection (after appendicectomy), internal midgut volvulus (intestinal obstruction), debilitating diarrhea, failure to thrive, 14 kg weight loss (despite TPN) | Monozygotic twin available, no immunosuppressive regimen | Living Donor Intestinal Transplantation with Interposition Arterial Graft and Duodeno-Colonic Anastomosis | Development of a Staphylococcus aureus right jugular septic thrombophlebitis just before central catheter removal | Syngeneic living-related intestinal transplantation between monozygotic twins. | none | none | none | - | - | - | - |
| Jaffe et al., 1997 | 10 cm of proximal jejunum remaining after retroperitoneal desmoid tumor resection, TPN-dependent for 21 months; acquired ganglioneuropathy causing dysmotility syndrome and pseudoobstruction, preventing material passage, with the terminal few centimeters resected (including the ileocecal valve), gastrostomy, and TPN-dependent for nearly 4 years | Family available, advantage of LR | Living Donor Jejunal Transplantation with Duodenojejunostomy and Jejunocolostomy | Distal ischemic necrosis, proximal anastomotic leak | Segmental Jejunal Resection with Vascular Preservation | - | 2 | 1,2 | - | 60,30 days | The patient lost a considerable amount of weight to a nadir of 37 kg, necessitating nutritional supplementation with TPN | Eating disorder |
| Cicalese et al., 2002 | Total enterectomies and intestinal failure (IF) due to trauma (gunshot wounds and motor vehicle accidents) | ABO compatible donor, Excellent HLA match | Living Donor Ileal Transplantation with Ileocolic Artery and Vein Anastomosis | None | Segmental Ileal Resection | none | none | none | - | - | All the recipients are currently alive and well | Liver dysfunction |
| Kim et al., 2012 | Extended total aganglionosis with resection of the entire colon and most of the small bowel, leaving only 50 cm of proximal jejunum, which was brought down to the anus in Duhamel fashion before small bowel transplant. | Parent Available | Living Donor Ileal Transplantation for Short Gut Syndrome (SGS) with Splenic Artery and Vein Anastomosis | None | Segmental Ileal Resection | none | 1 | 1 | - | - | The second mild attack of ACR, at 4 years after SBT | - |
| Asham et al., 2006 | Short gut syndrome from extensive resections due to familial polyposis, TPN-dependent for 2 years, failed rehab, lost 30 pounds | Family available | Isolated Living Donor Intestinal Transplant (LDITx) using an Ileocolic Pedicle Graft with End-to-Side Anastomosis to the inferior vena cava and distal aorta. | Pelvic hematoma | Segmental Ileal Resection | none | none | none | - | 11 days | The patient did well and went home on postoperative day 11. | - |
| Kuo et al., 1996 | Recurrent retroperitoneal desmoid tumor involving mesenteric artery and vein, causing intestinal obstruction, the patient refused chemotherapy, requiring prolonged TPN post-resection. | Monozygotic twin available | Isolated Living Donor Intestinal Transplant (LDITx) using an Ileocolic Segment Graft | Metabolic acidosis, patent arterial and venous anastomoses | Segmental Ileal Resection | - | none | none | - | - | - | - |
| Wu et al., 2021 | Short gut syndrome with TPN-related complications, including liver dysfunction, loss of central venous access, and catheter-related sepsis | Short Gut syndrome | Isolated Living Donor Intestinal Transplant (LDITx) using the Internal Iliac Vessel Technique | Vascular thrombosis, arterial stenosis, and pseudoaneurysms | Segmental Intestinal Graft Procurement with Internal Iliac Vessel Harvesting for Vascular Reconstruction | - | - | - | - | - | - | - |
| Beier et al., 2008 | Gastroschisis, volvulus, rejection of first small bowel graft, cholestasis, graft loss due to PTLD, megacystis microcolon, necrotizing enterocolitis. | Donor available | Isolated Living Donor Intestinal Transplant (LDITx) using a Distal Ileum Graft | - | Living-Related Small Bowel Procurement | - | - | - | - | - | - | - |
